# Current advances and future directions in the management of HBV-related hepatocellular carcinoma

**DOI:** 10.1093/gastro/goag060

**Published:** 2026-06-13

**Authors:** Andrew F Ibrahim, Julie Sang, Pojsakorn Danpanichkul, Kwanjit Duangsonk, Ju Dong Yang, Thomas A Kerr, Amit G Singal

**Affiliations:** School of Medicine, Texas Tech University Health Sciences Center, 3601 4th Street, Lubbock, TX 79430, USA; School of Medicine, Texas Tech University Health Sciences Center, 3601 4th Street, Lubbock, TX 79430, USA; Department of Internal Medicine, Texas Tech University Health Sciences Center, 3601 4th Street, Lubbock, TX 79430, USA; Department of Microbiology, Faculty of Medicine, Chiang Mai University, 110 Inthawarorot Road, Sri Phum, Mueang Chiang Mai, Chiang Mai 50200, Thailand; Karsh Division of Gastroenterology and Hepatology, Comprehensive Transplant Center, Samuel Oschin Comprehensive Cancer Institute, Cedars-Sinai Medical Center, 8700 Beverly Boulevard, Los Angeles, CA 90048, USA; Department of Internal Medicine, UT Southwestern Medical Center, 5323 Harry Hines Boulevard, Dallas, TX 75390-9030, USA; Department of Internal Medicine, UT Southwestern Medical Center, 5323 Harry Hines Boulevard, Dallas, TX 75390-9030, USA

**Keywords:** hepatitis B, hepatocellular carcinoma, antiviral therapy, immune checkpoint inhibitors

## Abstract

Hepatocellular carcinoma (HCC) remains a major driver of global cancer mortality, with HCC incidence projected to rise in the coming decades and chronic hepatitis B virus (HBV) continuing to account for a substantial proportion of cases worldwide. HBV-related HCC is distinguished by dual carcinogenic pathways: direct oncogenic effects mediated by viral persistence and genomic integration, and indirect carcinogenesis arising from chronic inflammation, fibrosis, and cirrhosis. Despite the success of nucleos(t)ide analogue (NA) therapy in suppressing HBV replication and reducing HCC incidence, residual risk persists, mandating risk-stratified surveillance even during long-term viral suppression. In parallel, advances in systemic therapy, particularly immune checkpoint inhibitor (ICI)-based combinations, have established immunotherapy-based regimens as preferred first-line options for unresectable disease. Emerging data have suggested a possible role for ICIs for select patients in earlier lines of disease, including the perioperative setting or in combination with transarterial chemoembolization for patients with intermediate-stage disease. This review synthesizes clinically relevant advances across the HBV-HCC continuum: molecular pathogenesis and risk stratification tools, antiviral strategies spanning curative and palliative settings, contemporary locoregional modalities, systemic regimens including ICI-based combinations, tyrosine kinase inhibitors, and biomarker-directed agents, and special clinical scenarios, such as portal vein tumor thrombus, high viral load, pregnancy, and viral coinfections. We emphasize HBV-specific safety considerations, including rigorous mitigation of reactivation risk with NA prophylaxis and standardized HBV DNA monitoring, and highlight future directions, such as validated composite biomarkers (e.g. circulating tumor DNA-based minimal residual disease assays), optimization of immuno-oncology locoregional therapy sequencing, and the potential influence of next-generation functional-cure HBV therapeutics on long-term HCC incidence and recurrence.

## Introduction

Hepatocellular carcinoma (HCC) is the third leading cause of cancer-related mortality worldwide, with an estimated 1.52 million new cases and 1.37 million deaths projected in 2050 [1]. HCC accounted for 3% of global cancer incidence and 5% of global cancer deaths [2]. Pandemic-related disruptions led to a worsening of HCC presentation and management. In Europe, significant stage migration was observed, with late-stage diagnoses (BCLC-D) more than doubling in Scotland and intermediate-stage (BCLC-B) cases reaching 50% in Romania [3, 4]. Concurrently, US data from 2020 to 2022 revealed a significant reduction in the likelihood of receiving curative treatment and a higher rate of non-treatment relative to the 2016–2019 era [5]. Although the contribution of liver cancer from steatotic liver disease is rising [6], hepatitis B virus (HBV) remains a major global driver of HCC and is estimated to account for 40%–50% of cases worldwide, with substantial heterogeneity and a particularly high contribution in East Asia and Sub-Saharan Africa [7–9]. Based on Global Burden of Disease estimates, the incidence of HBV-related HCC increased in 65 countries between 2000 and 2021 [10].

HBV vaccination programs have demonstrated substantial success in reducing HCC incidence in vaccinated populations. For example, Taiwan’s universal neonatal HBV vaccination program, initiated in 1984, was associated with a 35.9% reduction in HCC incidence among individuals aged 30 years or younger [11]. Long-term follow-up data from China further demonstrated that neonatal HBV vaccination conferred 84% protection against primary liver cancer over a 30-year period [12]. Despite implementation of neonatal vaccination campaigns and antiviral therapy programs, chronic HBV infection remains a leading risk factor for HCC, particularly in regions with high HBV prevalence, such as East Asia and sub-Saharan Africa [13, 14]. This review provides a comprehensive overview of the pathogenesis and current treatments for HBV-related HCC, emphasizing clinically relevant data and future directions.

## Pathogenesis and risk stratification

HBV promotes hepatocarcinogenesis through both direct viral oncogenic mechanisms and indirect effects mediated by chronic inflammation and fibrosis. Unlike other etiologies of HCC, HBV can drive malignant transformation even in the absence of cirrhosis [15]. Following infection, covalently closed circular DNA (cccDNA) persists in the nucleus of infected hepatocytes, enabling lifelong infection and persistent oncogenic signaling [16].

Although HBV DNA integration into the host genome is not required for viral replication, it represents a critical early oncogenic event. Integration occurs early during clonal tumor expansion and preferentially at sites of genomic fragility, leading to chromosomal rearrangements, insertional mutagenesis, and dysregulation of cancer-related genes [15, 17]. Integrated HBV DNA arises when linear double-stranded HBV DNA inserts into the host genome. Although these integrated sequences generally cannot generate a complete progeny virus, they can persist long term and continue to produce viral proteins, most notably hepatitis B surface antigen (HBsAg) and HBV X protein (HBx) [18]. High-throughput sequencing has shown that HBV integration events are more frequent in tumor tissue than in adjacent non-tumor liver and often localize within coding or regulatory promoter regions, supporting a direct role in hepatocarcinogenesis [19, 20]. Prognostic associations between HBV integration burden and outcome have been reported in selected sequencing-based resection cohorts. Péneau *et al.* using viral-capture sequencing in 177 HBV-related HCCs with paired non-tumor tissues, found that a high number of tumor HBV integrations was associated with poor prognosis independent of tumor size, microvascular invasion, differentiation status, and transcriptomic group [21]. Sung *et al.* similarly reported that, in 81 HBV-positive HCCs, the number of HBV integrations was associated with patient survival [22]. Nevertheless, these findings remain exploratory. Integration burden assessment is not yet standardized across platforms or bioinformatic pipelines, and current evidence largely derives from specialized research cohorts rather than from broadly validated clinical assays. Therefore, HBV integration burden should be regarded as an investigational prognostic correlate rather than an established clinical risk stratification marker.

A key downstream consequence of HBV integration is telomerase activation. The telomerase reverse transcriptase (TERT) promoter is the most recurrent hotspot for HBV DNA integration, occurring in 35%–40% of HBV-related HCC cases [19, 23]. Integration at this locus leads to increased telomerase expression, supporting cellular immortality, early clonal expansion, and aggressive tumor behavior. In addition, somatic TERT promoter mutations provide an alternative mechanism for telomerase activation, occurring in up to 60% of HCC cases overall [24]. Together, these alterations underscore telomere maintenance as a central oncogenic pathway in HBV-related HCC.

Viral proteins further contribute to direct oncogenesis. HBx and altered envelope proteins disrupt transcriptional regulation, cell-cycle control, and DNA damage responses. Prolonged HBx expression alters chromatin dynamics and induces epigenetic changes, including aberrant DNA methylation and histone acetylation, leading to silencing of tumor suppressor genes and promotion of malignant transformation [25, 26].

In parallel, chronic immune-mediated liver injury contributes substantially to HCC risk. Persistent antigen exposure during chronic HBV infection leads to repeated cycles of hepatocyte injury and regeneration, promoting fibrosis and increasing cumulative malignant risk over time [27]. Chronic infection is also associated with immune dysfunction, characterized by exhaustion of virus-specific CD8+ T cells and the development of an immunosuppressive hepatic microenvironment [28]. Compared with non-viral HCC, HBV-associated HCC tends to have a more immunosuppressive, immune-exhausted tumor microenvironment, characterized by increased cell death protein 1 (PD-1)-expressing regulatory T cells and a higher proportion of exhausted CD8+ resident memory T cells [29]. These changes impair immune surveillance and may influence tumor biology and treatment response.

## Antiviral therapy

### Primary prevention: long-term nucleos(t)ide therapy and reduction of incident HCC risk

Nucleos(t)ide analogues (NA) substantially reduce, but do not eliminate, HCC risk in chronic hepatitis B [30–34]. Entecavir and tenofovir are preferred first-line agents due to their greater antiviral potency and resistance barrier. Multiple large cohort studies and meta-analyses have demonstrated that NA therapy reduces HCC incidence compared with no treatment (Table 1). Importantly, even with long-term viral suppression, the risk of HCC persists [35–38]. In a meta-analysis by Tseng *et al.* comprising 34 studies, the 5-year cumulative incidence of HCC remained considerable in cirrhotic patients, ranging from 4.5% to 21.6% in those with compensated cirrhosis and 36.3% to 46.5% in those with decompensated cirrhosis. In non-cirrhotic patients, the 5-year incidence was lower but still significant, ranging from 0.5% to 6.9% [38].

**Table 1:** Effect of nucleos(t)ide analogue therapy on HCC risk compared with no treatment.

Study	Region	Study design	Antiviral therapy	Population	Follow-up	Key findings
Hosaka *et al.* [30]	Japan	Retrospective cohort study	Entecavir	472 entecavir-treated and 1,143 untreated HBV patients	5 years	5-yr HCC incidence 3.7% vs 13.7%; adjusted HR 0.37
Kumada *et al.* [31]	Japan	Retrospective cohort study	Lamivudine, adefovir, entecavir	117 matched pairs	10 years	NA therapy associated with reduced HCC risk (adjusted HR 0.28)
Wu *et al.* [32]	Taiwan	Retrospective cohort study	Lamivudine, telbivudine, entecavir	21,595 matched pairs	7 years	7-yr HCC incidence 7.3% vs 22.7%; adjusted HR 0.37
Wang *et al*. [33]	Taiwan	Retrospective cohort study	Lamivudine, telbivudine, entecavir, tenofovir	1,544 matched pairs	Variable	NA use significantly reduced the risk of HCC (HR 0.64) and overall mortality (HR 0.58)
Moriyama *et al.* [34]	Japan	Retrospective cohort study	Lamivudine, adefovir, entecavir, tenofovir	212 matched pairs	12.9 and 6.8 years in the NA and non-NA groups, respectively	NA did not affect HCC risk (HR, 0.68; 95% CI, 0.36-1.31; *P*= 0.25) after adjusting for other risk factors. NA use reduced the risk of HCC in cirrhotic patients (HR, 0.26; 95% CI, 0.08-0.85; *P*= 0.03).

CI, confidence interval; HBV, hepatitis B virus; HCC, hepatocellular carcinoma; HR, hazard ratio; NA, nucleos(t)ide analogue.

The comparative effectiveness of tenofovir versus entecavir for HCC prevention remains debated (Table 2). Several large retrospective studies and meta-analyses have suggested a modestly lower HCC risk with tenofovir [39–42], while others have demonstrated no significant difference between the two [43, 44]. The data are inconsistent, come exclusively from observational studies, and vary substantially by region and study methods. These studies reporting null findings state that observed advantages favoring tenofovir may be attributed to differences in baseline risk profiles and residual confounding. However, one newer study has associated tenofovir with a better prognosis and a significantly reduced mortality risk [39]. Since this remains an ongoing dilemma, and no prospective randomized trials have directly compared these agents for HCC prevention, current guidelines continue to view them as equally appropriate first-line options.

**Table 2:** Comparative studies of tenofovir versus entecavir on HCC risk and outcomes.

Study	Region	Design	Population	Key findings
Chung *et al.* [39]	Korea	Retrospective cohort study	6,525 HBV-HCC patients	Tenofovir improved OS vs entecavir (adjusted HR 0.79); stronger benefit after 2 yrs; effect greatest in early-stage disease
Dave *et al.* [40]	Multi-country	Systematic review and meta-analysis	14 studies, 263,947 person-years	Adjusted data favored TDF (ETV HR 1.27); no difference in cirrhotics
Choi *et al*. [41]	Multi-country	Systematic review and meta-analysis	15 studies, 61,787 CHB patients	TDF associated with 20% lower HCC risk vs entecavir (HR 0.80); effect persisted in cirrhosis and PS-matched cohorts; no difference in death/transplant
Yip *et al.* [42]	China	Retrospective cohort study	29,350 CHB patients	TDF associated with lower HCC risk vs entecavir (HR ∼0.36–0.39); effect persisted after PS matching
Tseng *et al.* [43]	Multi-country	Systematic review and meta-analysis	31 studies, 119,053 CHB patients	No significant difference between TDF and entecavir (adjusted HR 0.88)
Tan *et al.* [44]	Multi-country	Systematic review and meta-analysis	14 studies, 24,269 CHB patients	No significant difference between TDF and entecavir (HR 0.85)

CHB, chronic hepatitis B; ETV, entecavir; HBV, hepatitis B virus; HCC, hepatocellular carcinoma; HR, hazard ratio; OS, overall survival; PS, propensity score; TDF, tenofovir disoproxil fumarate; yrs, years.

### Tertiary prevention: reduction of recurrence after curative-intent therapy

Antiviral therapy has also been associated with a reduced risk for HCC recurrence after curative-intent treatments, such as surgical resection or ablation [45]. In a large nationwide cohort of 4,569 patients who underwent curative liver resection, postoperative NA therapy was independently associated with reduced recurrence and lower overall mortality [46]. Importantly, benefit has also been reported in patients with low baseline HBV DNA levels [47]. Biologically, this setting differs from primary prevention: early recurrence, typically within the first 2 years, is thought to reflect occult intrahepatic metastases or residual microscopic disease already present before treatment, whereas later recurrence more often represents de novo carcinogenesis in the remnant liver [48, 49]. By suppressing HBV replication, reducing hepatic inflammation, and preserving hepatic reserve, antiviral therapy may be particularly relevant to the latter process.

### Prophylaxis against HBV reactivation during locoregional or systemic therapy

In the absence of prophylaxis, HBV reactivation is common and associated with adverse outcomes. Prophylactic antiviral therapy significantly reduces the risk of reactivation across treatment modalities and is associated with improved survival [47]. A meta-analysis by Papatheodoridi *et al.* with 41 studies and 10,233 patients showed that HBV reactivation rates were lower in those who received HBV prophylaxis following HCC therapy: 0% versus 9% with ablation, 3% versus 20% with resection, 1% versus 23% with chemoembolization, and 3% versus 9% with systemic therapy [50]. Therefore, all HBsAg-positive patients undergoing locoregional or systemic therapy should start a high-barrier NA before therapy and continue throughout therapy and long-term thereafter, with HBV DNA and Alanine aminotransferase (ALT) monitoring every 3–6 months.

### HCC surveillance

Because antiviral therapy does not eliminate HCC risk, long-term surveillance remains mandatory in patients at elevated risk [51, 52]. The strongest evidence supporting surveillance comes from HBV populations. In a large randomized controlled trial from China, semiannual ultrasound and alpha-fetoprotein (AFP) screening was associated with increased detection of early-stage HCC and a 37% reduction in HCC-related mortality compared with no surveillance [53].

The American Association for the Study of Liver Diseases (AASLD) recommends HCC surveillance with ultrasound plus AFP every 6 months for patients with cirrhosis and for selected higher-risk non-cirrhotic patients with chronic hepatitis B. This approach applies to all transplant-eligible patients with cirrhosis, since detecting early-stage HCC can alter transplant priority [51]. In non-cirrhotic patients with chronic hepatitis B, surveillance is guided by demographic risk factors, including age, sex, race, and family history, and remains recommended for Asian men over 40 years, Asian women over 50 years, and African men over 40 years [51]. The 2023 HCC guidance further endorses formal risk stratification using validated clinical models, such as the PAGE-B and modified PAGE-B scores, which incorporate age, sex, and platelet count, to refine surveillance decisions [51]. While emerging biomarkers, including hepatitis B core-related antigen (HBcrAg), serum HBV RNA, AFP, and Protein Induced by Vitamin K Absence or Antagonist-II (PIVKA-II), may further refine risk assessment [54, 55], validated clinical scoring systems remain the foundation of surveillance decision-making in current practice.

Although surveillance is recommended for at-risk patients with chronic HBV infection and cirrhosis, several practical constraints limit its real-world effectiveness. Surveillance uptake remains suboptimal: a recent meta-analysis of 48 studies found that while approximately half of at-risk patients undergo some form of HCC surveillance, fewer than 10% receive the recommended biannual ultrasound, and utilization has not improved over time [56]. In addition, ultrasound sensitivity may be reduced in patients with obesity, advanced cirrhosis, or otherwise poor sonographic visualization [51, 57, 58]. Finally, substantial disparities in access to surveillance persist in many HBV-endemic and resource-limited settings, where limitations in infrastructure, trained personnel, and timely linkage to specialty care may hinder implementation of guideline-based screening [59].

### Curative and locoregional therapies

Management of HBV-related HCC generally follows established global treatment algorithms, as current evidence does not support etiology-specific modifications in treatment selection. Clinical decision-making is therefore primarily guided by tumor stage, hepatic functional reserve, and patient performance status, typically within the framework of the Barcelona Clinic Liver Cancer (BCLC) staging system [60].

Surgical resection is the treatment of choice for HCC patients with a solitary tumor, preserved liver function, and no portal hypertension, particularly in patients without cirrhosis [60]. This clinical profile is seen more frequently in HBV-related HCC, as carcinogenesis may occur in the absence of cirrhosis [15]. Increasing data, predominantly from Asia, suggest surgical resection can yield favorable outcomes in selected patients with limited multifocal disease or limited vascular invasion, although these extended criteria should likely only be pursued in expert centers after multidisciplinary discussion. Despite low perioperative mortality [61], recurrence remains the main cause of long-term death. For HBV-related HCC patients, high preoperative viral load is associated with a significantly increased recurrence rate following resection [62–64]. Such recurrences tend to appear within the first 2 years and are due to intrahepatic metastasis or residual microscopic disease [62, 65]. After curative resection or ablation, HCC recurs in 50%–70% of patients at 5 years across broad surgical cohorts encompassing all etiologies and tumor stages [48, 49]. Recurrence rates are higher in patients with adverse pathologic features: in the IMbrave050 trial, which enrolled patients with high-risk characteristics (tumor >5 cm, >3 tumors, microvascular or macrovascular invasion, or poor differentiation), recurrence exceeded 70% within 5 years [66]. Therefore, continued imaging follow-up with contrast-enhanced CT or MR imaging every 3–6 months during the first 2 years after resection, followed by longer intervals thereafter, is essential to detect recurrences.

Liver transplantation (LT) remains the definitive curative option for patients with early-stage HCC and underlying cirrhosis. Outcomes of LT for HBV-related HCC have improved dramatically in the antiviral era, with modern prophylaxis reducing HBV recurrence to <5% [67]. Survival after LT for HBV-related HCC is comparable to other etiologies when performed within accepted selection criteria. Bridging and downstaging strategies using locoregional therapy are commonly employed while awaiting transplantation.

For patients with unifocal HCC ≤3 cm who are not a surgical candidate or would require a major resection, thermal ablation (radiofrequency ablation or microwave ablation) is an alternative first-line option and is regarded as a potentially curative treatment [51]. If the tumor is >3 cm or is in an unfavorable location (e.g. adjacent to critical structures), other liver-directed options can be used, including radiation segmentectomy (targeted radioembolization), external beam radiation therapy, or transarterial chemoembolization (TACE) (sometimes combined with ablation for 3–5 cm lesions). Radiation-based approaches are especially useful when proximity to vital anatomy limits safe ablation [68].

Beyond the curative setting, TACE remains the standard treatment for intermediate-stage (BCLC-B) HCC and is widely used in HBV-endemic regions. In HBV-related cohorts, survival after TACE is highly heterogeneous, with reported median overall survival (OS) ranging from 12 months to over 40 months depending on tumor burden and hepatic reserve [69]. Patients receiving nucleoside analogue therapy also demonstrated significantly improved outcomes with 5-year survival rates of approximately 40% compared to 14% without antiviral treatment [70]. The role of adjuvant TACE after resection remains controversial. Several Chinese studies in high-risk HBV-related HCC populations have reported improved recurrence-free and OS with postoperative TACE, but these findings have not been consistently reproduced in broader populations, and the strategy is not routinely endorsed by Western guidelines [71, 72]. These approaches are discussed further in the neoadjuvant and adjuvant therapy section below.

### Systemic therapies

The treatment landscape for advanced HCC has been transformed by immune checkpoint inhibitor (ICI)-based combinations, which are now the preferred first-line approach. The IMbrave150 trial demonstrated that atezolizumab plus bevacizumab has superior OS compared to sorafenib (median 19.2 versus 13.4 months; hazard ratio [HR] 0.66), with a confirmed objective response rate of 29.8% and median progression-free survival (PFS) of 6.9 months [73]. The combination also achieved quality-of-life benefits, with a median time to deterioration of 11.2 months versus 3.6 months with sorafenib [74]. Durvalumab plus tremelimumab (STRIDE regimen) offers an alternative first-line option, particularly for patients with contraindications to vascular endothelial growth factor (VEGF)-inhibiting therapy [75]. More recently, CheckMate 9DW demonstrated superior OS with nivolumab plus ipilimumab compared with sorafenib or lenvatinib (median 23.7 months [95% CI 18.8–29.4] vs 20.6 months [17.5–22.5]), further expanding first-line ICI options [76]. The CARES-310 trial similarly showed improved survival with camrelizumab plus rivoceranib compared with sorafenib in a predominantly HBV-endemic population [77]. Although each regimen has been shown to be efficacious compared to tyrosine kinase inhibitors (TKI) therapy, there are no direct head-to-head data comparing ICI combinations and cross-trial comparisons are discouraged given differences in the patient populations [78, 79].

Sequential therapy strategies after first-line immunotherapy remain an evolving area, with TKI representing the preferred second-line approach. Sorafenib or lenvatinib are commonly used in this setting, with data suggesting favorable outcomes with lenvatinib following atezolizumab plus bevacizumab [80, 81]. Lenvatinib is approved for first-line treatment of unresectable HCC, not specifically as post-immunotherapy second-line therapy. Thus, use after atezolizumab plus bevacizumab is off-label and guided by recommendations and emerging data rather than indication-specific regulatory approval [82]. Cabozantinib and regorafenib are established options after prior TKI therapy, while ramucirumab is indicated for patients with AFP levels ≥400 ng/mL [83]. Dual immune checkpoint blockade with nivolumab plus ipilimumab has also demonstrated promising activity in selected patients [51]. Emerging multicenter retrospective data demonstrate retained efficacy even after prior PD(L)1 exposure, with an objective response rate of 22%, supporting its use as a salvage option in later-line therapy [76, 84].

HBV infection does not appear to negatively impact the efficacy of immunotherapy, with meta-analyses suggesting potential improved tumor response rates compared to non-viral HCC [85]. However, preservation of liver function is critical for patients undergoing systemic therapy, as many die of liver dysfunction and not HCC [86]. Indeed, high baseline HBV DNA load (>500 IU/mL) is associated with worse survival outcomes in patients receiving immunotherapy, whereas patients with undetectable baseline HBV DNA demonstrate better PFS [85, 87]. Recent data suggest that combining ICIs with antiviral therapy yields dual benefits, including lowering HBV markers and improving prognosis, with virological responders showing substantially better survival (HRs 1.64–8.06) [88].

Whether etiology should influence systemic therapy selection remains an open question. Although HBV-related HCC may differ biologically from nonviral HCC, immunogenomic analyses suggest that therapeutic responsiveness may be better captured by inflamed versus non-inflamed tumor classes than by etiology alone [89]. In this regard, Montironi *et al.* showed that HCC can be categorized into inflamed and non-inflamed immune classes with distinct implications for immunotherapy response, supporting future trial stratification by both viral status and molecular immune phenotype. At present, however, etiology alone is insufficient to justify differential therapeutic selection in routine practice.

In addition, whether clinical outcomes differ by underlying liver disease etiology remains incompletely defined. Several phase 3 trials of ICIs, including IMbrave150, HIMALAYA, and CheckMate 9DW, enrolled mixed-etiology populations and were not powered for etiology-specific comparisons [66, 74, 76, 90]. While unadjusted subgroup analyses from IMbrave150 suggested lower efficacy in patients with non-viral etiologies, subsequent adjusted analyses accounting for baseline differences showed no statistically significant difference in treatment effect by etiology, supporting broadly consistent efficacy across HBV, HCV, and non-viral HCC [73, 91]. These findings may reflect underlying immune biology, as chronic viral antigen exposure in HBV-related HCC is associated with T-cell exhaustion and an immunosuppressive tumor microenvironment, whereas non-viral HCC, particularly metabolic dysfunction-associated steatotic liver disease (MASLD)-related disease, has been associated with impaired antitumor immune surveillance and reduced responsiveness to immunotherapy in preclinical and translational studies [92, 93]. Accordingly, future trials could consider stratification by viral status and integration of molecular immune profiling to better define subgroups most likely to benefit from immunotherapy.

## Combination and integration therapies

### Immune checkpoint inhibitors and locoregional therapy

The integration of systemic therapy with locoregional treatments (LRT) represents a pivotal evolution in the management of unresectable, liver-localized HCC and is particularly relevant for HBV-associated HCC. Mechanistically, HBV-related hepatocarcinogenesis is characterized by chronic antigenic stimulation, intrahepatic immune exhaustion, and a highly tolerogenic tumor microenvironment, features that may render HBV-HCC particularly amenable to immune modulation [94]. LRTs, such as TACE, cause substantial tumor necrosis and can promote immunogenic cell death, leading to the release of tumor-associated antigens and exposure of damage-associated molecular patterns (DAMPs) [95, 96]. However, the biology is not purely immunostimulatory. In parallel with this antigenic “priming,” TACE generates a hypoxic tumor microenvironment that increases hypoxia-inducible factor 1-alpha (HIF-1α) signaling and upregulates vascular endothelial growth factor (VEGF) and programmed death ligand-1 (PD-L1), fostering recruitment of immunosuppressive populations (including regulatory T cells and myeloid-derived suppressor cells) and impairing dendritic cell function [97]. This sequence, early immune activation followed by compensatory immunosuppression, forms a key mechanistic rationale for pairing TACE with anti-VEGF agents and ICIs [97]. In HBV-infected livers, these treatment-induced antigenic cues occur in the setting of chronic viral antigen exposure and adaptive immune dysfunction, which may further shape immune priming in transplant-eligible patients and potentially enhance responsiveness to immune checkpoint inhibition [98].

Recent phase 3 data clinically validate this strategy (Table 3). The phase 3 EMERALD-1 trial provided high-level evidence for this approach, demonstrating that TACE combined with durvalumab and bevacizumab significantly improved progression-free survival compared with TACE alone in patients with unresectable HCC amenable to embolization; however, OS data remain immature for EMERALD-1 [99]. Similarly, the LEAP-012 trial showed a significant improvement in PFS with the addition of lenvatinib and pembrolizumab to TACE, but did not show a statistically significant OS benefit at the interim analysis [100]. Consistent with these findings, the recently reported TALENTACE study evaluating TACE combined with atezolizumab and bevacizumab also demonstrated a significant improvement in PFS, further supporting the concept that the addition of immunotherapy and VEGF inhibition to TACE can delay disease progression; however, similar to the EMERALD-1 and LEAP-012 trials, OS for TALENTACE remained immature at the time of the interim analysis, with no statistically significant difference yet observed between the combination arm and TACE alone (HR 0.96) [101]. Given that OS remains one of the most clinically meaningful endpoints in HCC, these regimens cannot yet be considered practice-changing standards of care. While the consistent PFS improvements support the biologic rationale for combining locoregional and systemic therapy, confirmation of a clear and durable OS benefit will be essential before routine adoption into standard clinical practice.

**Table 3:** Key clinical trials of combination and integration therapies relevant to HBV-related HCC.

Study	Phase/design	Intervention arm vs. control	Population	Key findings
**EMERALD-1** (Sangro *et al.* [99])	Phase 3, Randomized, Double-blind	TACE + Durvalumab + Bevacizumab vs. TACE + Placebo	Unresectable, liver-confined HCC. 50% HBV+	**Primary Endpoint Met:** Median PFS improved (15.0 vs 8.2 months; HR 0.77). Demonstrates benefit of integrating TACE + IO + VEGF inhibition. **OS**: data immature.
**LEAP-012** (Kudo *et al.* [100])	Phase 3, Randomized, Double-blind	TACE + Lenvatinib + Pembrolizumab vs. TACE + placebo	Unresectable HCC amenable to TACE	**Primary Endpoint Met:** Median PFS improved (14.6 vs 10.0 months; HR 0.66). **OS:** no statistically significant benefit at interim analysis (HR 0.80).
**TALENTACE** (Dong *et al*. [101])	Phase 3, Randomized, Open-label	On-demand TACE + Atezolizumab + Bevacizumab vs. TACE alone	Unresectable, liver-confined HCC. High tumor burden. 80% HBV+.	**Primary Endpoint Met:** Median PFS improved (11.3 vs 7.0 months; HR 0.71). **OS:** data immature (HR 0.96).
**TACTICS** (Kudo *et al*. [102])	Phase 2, Randomized, Open-label	TACE + Sorafenib vs. TACE alone	Unresectable HCC amenable to TACE	**Primary Endpoint Met:** Median PFS (time to unTACEable progression) improved (25.2 vs 13.5 months; HR 0.59). **OS:** No significant difference (36.2 vs 30.8 months; HR 0.86).
**LAUNCH** (Peng *et al*. [103])	Phase 3, Randomized, Open-label	Lenvatinib + TACE vs. Lenvatinib monotherapy	Advanced HCC; >85% HBV+	**Primary Endpoint Met:** Median PFS improved (10.6 vs 6.4 months; HR 0.43). **OS: Median OS i**mproved (17.8 vs 11.5 months; HR 0.45).
**IMbrave050** (Qin *et al*. [66])	Phase 3, Randomized, Open-label	Atezolizumab + Bevacizumab vs. Active Surveillance	High-risk recurrence after curative resection or ablation (HBV-predominant)	**Primary Endpoint Met**: Recurrence-free survival improved (HR 0.72). **OS:** data immature.
**CARES-009** (Wang *et al.* [104])	Phase 2/3, Randomized, Open-label	Perioperative camrelizumab + rivoceranib vs. surgery alone	Resectable HCC at intermediate or high risk of recurrence (HBV-predominant population)	**Primary Endpoint Met:** Recurrence-free/event-free survival improved (HR 0.52). **OS:** data immature.

HBV, hepatitis B virus; HCC, hepatocellular carcinoma; HR, hazard ratio; IO, immuno-oncology; OS, overall survival; PFS, progression-free survival; TACE, transarterial chemoembolization; VEGF, vascular endothelial growth factor.

The above findings highlight a broader methodological challenge in HCC, namely, whether PFS is an adequate surrogate for clinically meaningful outcomes. In cirrhotic patients, radiologic progression may not directly translate into worsened survival, as prognosis is frequently driven not only by tumor burden but also by underlying liver function and risk of hepatic decompensation [105]. In addition, locoregional therapies, such as TACE, can alter imaging characteristics without necessarily reflecting true biologic progression, and treatment strategies often permit retreatment beyond conventional radiologic progression [106]. As a result, improvements in PFS may not consistently correlate with OS or patient-centered outcomes. These limitations underscore the importance of interpreting PFS endpoints with caution in this setting and reinforce the need for trials demonstrating durable OS benefit.

In patients with HBV-related HCC and portal vein tumor thrombus (PVTT), immunotherapy-based systemic therapy also represents an important treatment option. A dedicated Vp4 PVTT subanalysis of IMbrave150 demonstrated a numeric OS benefit with a manageable safety profile, suggesting feasibility even in patients with main portal trunk invasion in HBV-predominant cohorts [107].

### TACE and tyrosine kinase inhibitors

Beyond immunotherapy, the combination of TACE with TKIs could theoretically address the surge in VEGF and HIF-1α that typically follows embolization, which can otherwise drive revascularization and tumor recurrence [108, 109]. However, randomized trials of TACE + TKI have produced inconsistent clinical benefit. Large studies adding sorafenib to TACE, such as SPACE and TACE-2, did not demonstrate clinically meaningful improvement in progression endpoints [110, 111]. The phase 2 TACTICS trial reported improved PFS and prolonged time to “unTACEable progression”, but did not show a statistically significant OS benefit [102]. More recently, the phase 3 LAUNCH trial in advanced HCC reported improved OS and PFS with lenvatinib + TACE versus lenvatinib alone, but its generalizability across different practice settings and stages remains uncertain [103]. Consistent with this mixed evidence base, the Barcelona Clinic Liver Cancer framework states that TACE-based combinations should not be recommended outside clinical trials, and therefore TACE–TKI strategies are not routinely endorsed as standard care [112].

### Bridging and downstaging to transplant

Systemic therapy serves a critical role in “conversion therapy”, aiming to downstage unresectable patients to eligibility for curative interventions such as surgical resection or LT [98]. Successful downstaging of patients to within Milan criteria (single tumor ≤5 cm or up to 3 tumors ≤3 cm) is associated with excellent long-term outcomes [113]. One study indicates that patients who are successfully downstaged achieve a 5-year post-transplant survival rate comparable to that of patients who met transplant criteria ab initio [114]. In the context of HBV, maintaining strict viral suppression with potent NAs during this bridging period is paramount to prevent graft reinfection and ensure that the liver reserve remains adequate for major surgical interventions [115].

More recently, ICIs have been used as bridging/downstaging therapy in carefully selected LT candidates, but pre-transplant exposure is associated with a measurable risk of post-LT allograft rejection, with risk appearing inversely related to the ICI washout interval [116]. Contemporary cohort and individual patient–level analyses suggest that longer washout periods, such as >50 days and often >3 months, may reduce rejection risk toward that seen in ICI-unexposed recipients, supporting close coordination with transplant teams when ICIs are used pre-LT [117].

Beyond downstaging and bridging strategies, emerging perioperative systemic approaches are beginning to reshape the curative-intent setting in HCC. In the phase 3 IMbrave050 trial, adjuvant atezolizumab plus bevacizumab significantly improved recurrence-free survival compared with active surveillance in patients at high risk of recurrence after curative resection or ablation, representing the first positive adjuvant immunotherapy trial in HCC [66]. However, one updated study revealed that this benefit was not statistically significant compared with active surveillance, raising uncertainty regarding the durability and clinical significance of this approach [118]. Accordingly, adjuvant immunotherapy should still be considered investigational and has not yet been established as a standard of care. Further studies with longer follow-up are needed to determine whether a meaningful OS benefit can be achieved and to better define optimal patient selection.

More recently, the CARES-009 study further extended the use of systemic immunotherapy in the perioperative setting, demonstrating improved recurrence-free (or event-free) survival with systemic therapy administered around surgical resection compared with surgery alone [104]. Together, these data suggest a potential role for perioperative systemic therapy to reduce post-curative recurrence, including in HBV-predominant populations, although further validation, including OS outcomes, is needed.

## Special scenarios

### High viral load and acute hepatitis flares

A unique challenge in HBV-HCC is the presentation of malignancy concurrent with an acute hepatitis flare [119]. In these scenarios, the immediate initiation of oncologic therapy, especially TACE or hepatic resection, can precipitate acute-on-chronic liver failure [120]. The primary goal must be to stabilize liver function through the immediate administration of potent NAs [121]. Oncologic interventions should generally be deferred until the ALT improves and liver function stabilizes, often requiring a delay of several weeks [119]. However, once the flare has resolved, systemic therapy or LRT should not be withheld, provided the patient remains on continuous antivirals to prevent reactivation [122]. Nevertheless, any decision to postpone therapy should be made through multidisciplinary review, weighing the need for timely cancer control against the risk of precipitating hepatic decompensation.

### Pregnancy and reproductive age

As HBV-related HCC can occur in younger adults, management during pregnancy or in patients of reproductive age requires specialized care [123]. In pregnant patients diagnosed with HCC, a multidisciplinary approach is essential. Tenofovir disoproxil fumarate (TDF) is the preferred antiviral due to its established safety profile in pregnancy and efficacy in preventing vertical transmission [124]. Systemic therapies, particularly VEGF inhibitors and ICIs, are generally avoided during pregnancy due to teratogenic risks [123]. In young adults, fertility preservation counseling should be offered prior to the initiation of systemic anticancer therapy or radiotherapy [125]. Furthermore, cascade screening of family members is critical in these cases to identify other carriers and initiate surveillance early [126].

### Coinfections (HDV and HIV)

Coinfection with hepatitis D virus (HDV) accelerates fibrosis progression and increases HCC risk compared to HBV mono-infection [127]. HDV coinfection is associated with faster fibrosis progression, conferring an almost fourfold higher risk of decompensated cirrhosis (RR 3.82) and liver-related mortality (RR 3.78) compared with HBV monoinfection [128]. The recent approval of bulevirtide, a viral entry inhibitor, provides a new tool for virologic control [129]. Phase 3 trials have demonstrated significant reductions in HDV RNA and ALT normalization, though its long-term impact on HCC incidence remains under investigation [129]. In patients coinfected with human immunodeficiency virus (HIV), the interactions between antiretroviral therapy (ART) and oncologic agents must be carefully managed. Regimens should include tenofovir (TDF or tenofovir alafenamide) to suppress HBV, and care must be taken to avoid drug-drug interactions. With sustained viral suppression and immune reconstitution on ART, cohorts have reported comparable HCC treatment outcomes in HIV/HBV coinfected patients relative to mono-infected controls, although results remain heterogeneous across studies [130].

## Emerging therapies

### HBV-targeted cellular therapies

The persistence of HBV antigens on the surface of HCC cells makes them attractive targets for cellular immunotherapy. Chimeric antigen receptor-T cells and T-cell receptor-T cells engineered to recognize HBsAg or viral peptides are currently in early-phase clinical trials [131]. These “living drugs” aim to eliminate HBsAg-positive tumor cells and potentially clear the reservoir of infected hepatocytes [132]. While preclinical data are promising, a major challenge is “on-target, off-tumor” toxicity, where the engineered T cells may attack non-malignant, HBV-infected hepatocytes, leading to severe hepatitis [132, 133]. Strategies to tune the affinity of these receptors are being explored to mitigate this risk.

### Toward functional cure

Achieving a functional cure, defined as the sustained loss of HBsAg, is the ultimate goal of HBV therapeutics. Novel agents, such as antisense oligonucleotides (ASO) and small interfering RNAs (siRNAs), are designed to degrade viral RNA, thereby reducing antigen burden and potentially functionally suppressing transcription from cccDNA [134]. In the phase 2 b B-CLEAR trials, the ASO bepirovirsen achieved sustained HBsAg and HBV DNA loss in 9%–10% of treated patients [135]. By alleviating immune exhaustion induced by chronic antigen exposure, these agents may restore host immune surveillance. However, the long-term impact of sustained HBsAg loss on HCC risk remains uncertain. HBV DNA integration into the host genome can persist even after HBsAg loss and may continue to exert oncogenic effects [136]. Therefore, while novel agents that achieve functional cure are promising, it is not yet clear whether they will translate into a meaningful reduction in HCC incidence or recurrence beyond what is observed with current NAs [137]. Further longitudinal studies are needed to clarify whether HBsAg loss achieved through these approaches confers additional oncologic benefit.

### Precision oncology and liquid biopsy

Circulating tumor DNA (ctDNA) is emerging as a powerful tool for precision oncology in HBV-HCC [138]. In HBV-related cancers, the detection of HBV-host fusion sequences or specific mutational signatures in plasma can serve as a highly specific biomarker for minimal residual disease post-resection [139, 140]. Integrating ctDNA dynamics into clinical decision-making could help identify patients who would benefit from adjuvant therapy or intensified surveillance before radiological recurrence becomes evident.

## Future directions and conclusions

The landscape of HBV-HCC management is poised for further transformation. The greatest long-term reductions in HBV-related HCC incidence and mortality will likely continue to come from broad implementation of universal HBV vaccination, expanded diagnosis and linkage to care, and durable viral suppression with NAs, coupled with risk-stratified surveillance to enable curative detection [141, 142]. Despite advances in systemic therapy, optimizing curative-intent strategies remains a priority. Although adjuvant immunotherapy after resection/ablation has shown mixed results and has not yet established a consistent standard, perioperative systemic approaches such as CARES-009 demonstrate that integrating systemic therapy around surgery can improve recurrence-free/event-free outcomes in HBV-predominant cohorts and warrant further validation and refinement [104]. Further studies are needed to clarify the role of ICIs in earlier lines of disease and to develop standardized frameworks for combining locoregional therapy with immunotherapy, with focus on timing, sequencing, endpoints, and patient selection.

In advanced/unresectable disease, major society guidance supports both anti-PD-(L)1 plus anti-VEGF therapy, such as atezolizumab–bevacizumab, and dual immune checkpoint blockade, such as durvalumab–tremelimumab, as preferred first-line strategies, with regimen choice individualized based on contraindications and safety considerations [51]. Ultimately, continued progress in HBV-related HCC will depend on integrating effective oncologic therapy with rigorous, long-term HBV control through multidisciplinary care pathways and scalable public-health programs.

## Data Availability

No new datasets were generated or analyzed for this review.
